# Connectomics of Bone to Brain—Probing Physical Renderings of Cellular Experience

**DOI:** 10.3389/fphys.2021.647603

**Published:** 2021-07-12

**Authors:** Melissa L. Knothe Tate, Abhilash Srikantha, Christian Wojek, Dirk Zeidler

**Affiliations:** ^1^Blue Mountains World Interdisciplinary Innovation Institute, Sydney, NSW, Australia; ^2^Corporate Research and Technology, Carl Zeiss AG, Oberkochen, Germany; ^3^Carl Zeiss MultiSEM GmbH, Oberkochen, Germany

**Keywords:** connectomics, imaging, machine learning, cell, cell memory, cellular epidemiology

## Abstract

“Brainless” cells, the living constituents inhabiting all biological materials, exhibit remarkably *smart*, i.e., stimuli-responsive and adaptive, behavior. The emergent spatial and temporal patterns of adaptation, observed as changes in cellular connectivity and tissue remodeling by cells, underpin neuroplasticity, muscle memory, immunological imprinting, and sentience itself, in diverse physiological systems from brain to bone. Connectomics addresses the direct connectivity of cells and cells’ adaptation to dynamic environments through manufacture of extracellular matrix, forming tissues and architectures comprising interacting organs and systems of organisms. There is imperative to understand the physical renderings of cellular experience throughout life, from the time of emergence, to growth, adaptation and aging-associated degeneration of tissues. Here we address this need through development of technological approaches that incorporate cross length scale (nm to m) structural data, acquired via multibeam scanning electron microscopy, with machine learning and information transfer using network modeling approaches. This pilot case study uses cutting edge imaging methods for nano- to meso-scale study of cellular inhabitants within human hip tissue resected during the normal course of hip replacement surgery. We discuss the technical approach and workflow and identify the resulting opportunities as well as pitfalls to avoid, delineating a path for cellular connectomics studies in diverse tissue/organ environments and their interactions within organisms and across species. Finally, we discuss the implications of the outlined approach for neuromechanics and the control of physical behavior and neuromuscular training.

## Introduction

Cells of the human body populate their habitat through division, starting with two cells at conception and expanding to over 70 trillion cells over the course of a lifetime (Knothe Tate, 2017). Throughout the lifespan of the organism they inhabit, cells memorialize the biophysical and chemical stimuli they experience via gene expression of structural proteins created from molecular building blocks, e.g., amino acids. In this way, cells encode an organism’s and their own experiences in the physical world, by creating and adapting tissues, throughout life. Just as punch cards encode the recursive logic of textile weaves created with weaving looms (where card holes allow passage of hooks and the fibers they shuttle), genes encode and translate the arrangement of amino acids comprising elastin, collagen and other structural proteins making up tissue weaves (Knothe Tate, 2017, 2020; Ng et al., 2017a,b). A major barrier to understanding the emergent behavior that underpins this tissue genesis and adaptation is the lack of methods to image and analyze cellular connectivity across length and time scales.

The manuscript proposes a paradigm shifting approach to understand the cellular underpinnings of diseases as different as osteoarthritis and early onset dementia in bone and brain. We know as biologists that cells manufacture, remodel and adapt tissues throughout life (Knothe Tate et al., 2016a; Putra et al., 2019). The tissues render physically the collective cellular experience, reflected in architectures (bones) and memories (brain) which themselves exhibit *emergent properties* (Knothe Tate, 2020). These emergent properties cannot simply be deduced from the individual parts, which themselves do not exhibit such properties; rather, these emergent properties arise from spatial and temporal arrangements among multiple parts, e.g., memories that are physically encoded in neurons are not observable in single neurons but rather emerge from the spatial arrangement and temporal behavior of interacting neurons in the brain. A pathological example of emergence would be disease emergence, e.g., of osteoarthritis in the musculoskeletal system or early onset dementia in the brain, which cannot be predicted based on the occurrence of a single sick cell but rather at the stage of loss in function or loss in return to homeostasis due to emergence of disease amongst groups of cells that interact.

The elucidation of such disease emergence represents a currently untenable yet compelling research problem. On the one hand, the lack of methods to probe and understand emergent behavior of inhabitant cells within their complex ecosystems presents a hurdle to understanding and fundamental discoveries. On the other hand, the role of cell populations and the loss of their connectivity in disease progression has been stymied by the tradeoff between achieving sufficient resolution across vastly different length and time scales, e.g., single field of view and single time point images (nano- to microscale for electron to optical microscopy), and other imaging modalities that enable high temporal albeit less spatial resolution (MRI). Rapid advances in the field seek to overcome this current hurdle. To address each of these points, workflows are needed to render and analyze vast amounts of imaging data from nano- to meso- length scales. The manuscript describes that process and sets a path forward.

The neuroscience community refers to the totality of cellular connections and their three-dimensional (3D) networks, e.*g.*, in the brain, as the *connectome* and the process of rendering, analyzing and understanding the connectome as *connectomics* (Seung, 2012; Jbabdi and Behrens, 2013; Blakely, 2021). A recently integrated biosystems engineering, imaging and analysis platform enables a connectomics approach to map cellular connectivity across organs as diverse as brain and bone (Eberle et al., 2015; Knothe Tate et al., 2016c, 2019; Pereira et al., 2016). Tested in mouse brains (Mikula et al., 2012; Lichtman et al., 2014; Hayworth et al., 2015; Mikula and Denk, 2015; Swanson and Lichtman, 2016; Hayworth et al., 2020; Günther et al., 2021) and in our own pilot studies of the human hip (Eberle et al., 2015; Pereira et al., 2016; Knothe Tate, 2017), as well as validated through the delineation of standardized protocols and workflows (Ngo et al., 2019), these biosystems engineering approaches may find future applications relevant for every organ of the body.

**FIGURE 1:**
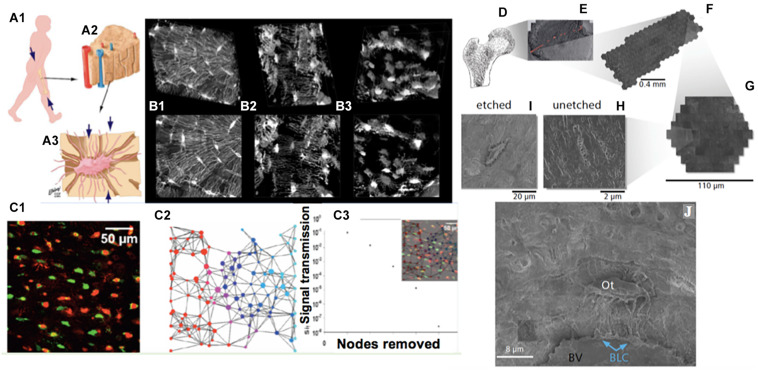
Imaging cellular networks and environments across length scales, from nano to meso, using cross length scale imaging (multi-beam scanning electron microscopy) of the cellular inhabitants of the human femoral neck, i.e., osteocytes, as a case study. **(A)** Organism **(A1)** to tissue **(A2)** to cellular (osteocyte, **A3**) length scales demonstrating the most prevalent cellular inhabitants of bone, osteocytes. During development, cells manufacture the tissues comprising the femoral head and neck (proximal femur, **A1,D**); the cellular inhabitants of bone, cartilage and other tissues model (during growth) and remodel (enabling adaptation) the respective tissues of their local environment through up- and down-regulation of structural protein transcription, and secretion into the extracellular matrix. **(B)** The cellular network of bone’s resident osteocytes changes throughout life, in health and disease (**B1**: healthy, **B2,B3**: diseased). **(B,C)** The loss in network connectivity reflects the health status of the cells (**C1**, **live** and **dead** osteocytes visualized using an ethidium bromide assay) as well as the patency of the network (**C2**—stochastic network model with nodes representing cells**, C3**—calculation of loss in information transfer with loss in network nodes); loss of viable cells within the network results in loss in network connectivity and subsequent diminished information transfer capacity across and within the network. **(D–J)** mSEM imaging, combined with image stitching and Google Maps API geonavigation applications, enables high resolution imaging of inhabitant cells within tissues, as well navigation and analysis of single cells and their complex networks, seamlessly across length scales (**D,** femoral head and neck; **E,** section through the femoral head created by stitching together of many images, comprising arrays; **F**, of hexagons; **G**, themselves made of arrays of electron beams). Rather than using single electron beams as in traditional electron microscopy, mSEM uses arrays of 61 and more beams **(F,G)** to capture large areas of tissue (mesoscale, **E**) with nanoscale resolution **(H–J)**. Through inorganic and organic etching procedures adapted from atomic force microscopy, the third dimension of cellular networks may be captured **(H,I)** and the local and global environment of tissues’ cellular inhabitants (Ot, Osteocyte; BLC, Bone Lining Cell) can be explored within tissue contexts (BV, upper half of oval Blood Vessel, above which bony matrix is seen). Images adapted and used with permission (**A,** Knothe Tate et al., 2010; **B,** Knothe Tate et al., 2002; **C,** Anderson et al., 2008; **D–J,** Knothe Tate et al., 2016c).

Here, key enabling steps are described for quantifying relationships and connectivity between cells in different disease states. Specifically, we test machine learning algorithms with cellular network maps of the human hip to elucidate the role of cell networks in organ and organism (patho)physiology throughout life (Figure 1). This approach may pave the way for next generation theranostics, i.e., enabling prediction of emergent cell scale pathology, including disease detection as well as treatment, well before permanent damage occurs at tissue and organ length scales. Based on the results of this pilot study, we assess opportunities and identify potential pitfalls of the integrated imaging, modeling and machine learning approaches.

### Datasets Rendered as Cellular Networks in Maps of Human Tissue

Human tissue samples from the femoral neck and head of patients undergoing total hip replacement were obtained with Institutional Review Board Approval (Cleveland Clinic IRB12-335). Samples were prepared for electron microscopy (EM) using a published protocol (Ngo et al., 2019) developed to enable fixation and polymethyl methacrylate embedding and electron microscopy of mesoscopic samples which exceed the typical diffusion path lengths for mm-sized electron microscopy samples.

Samples were first etched (to expose cells just below the block face surface) and imaged using multi-beam Scanning Electron Microscopy, to achieve nano- to meso-scale renderings of cellular inhabitants (mainly osteocytes with some red blood cells visible in resulting images). Based on this protocol, carbon coating provided sufficient contrast to visualize osteocytes exposed by chemical etching on the surface of the sample block. Although not used for the current study, sequential layers of cellular networks could be revealed by reiterating the etching and imaging steps, resulting in a volume of tissue with fully rendered three dimensional (3D) cellular network.

Three datasets were acquired using three generations of multibeam Scanning Electron Microscopes (mSEM) to image three different samples, starting with a 61 beam prototype at 12 nm pixel size and ending with a state-of-the-art commercial system (Zeiss mSEM 505) (Table 1).

**TABLE 1:** Dataset metrics from three generations of mSEM maps from three different human hip samples obtained with IRB approval.

**Data metrics**	**1st generation (gen)^#^**	**2nd gen^+^**	**3rd gen**
Total area imaged (mm^2^)	5.69	13.1	1,810
Total images in area	54,717	100,589	7,335,982
Multibeam FOVs	897	1649	120,262
Pixels (megapixels)	75,276	857,086	1.07 × 10^10^
Size (Terabytes)	0.08	0.87	10.98

*FOVs refers to Fields of View. See links for navigable, rendered maps comprising each dataset:*

*^#^https://www.mechbio.org/sites/mechbio/files/maps5/index.html (Knothe Tate et al., 2016c).*

*^+^https://www.mechbio.org/sites/mechbio/files/maps7/index.html user: mechbio, password: #google-maps.*

In our first pilot study (Knothe Tate et al., 2016c; Pereira et al., 2016), we tested the feasibility of using the Google Maps API platform to stitch and render the maps in a way that would be accessible to, as well as navigable and quantitatively analyzable, by scientists and the lay public alike. The resulting data set was annotated using Google Maps’ pins to mark manually viable and *pyknotic* cells (necrotic and apoptotic cells are typically identified by condensation of the chromatin and fragmentation of the nucleus, defining *pyknotic*). As a surrogate identification factor (classifier for machine learning implementation), osteocytes with less than three visible processes were identified visually and manually pinned as *pyknotic*, indicated by a red pin. Osteocytes with more than three visible processes were identified visually and manually pinned as *viable*, indicated by a green pin. The manual process took several weeks for the 1st generation dataset (Table 1). Full details of this process are described in previous publications (Knothe Tate et al., 2016c; Pereira et al., 2016).

### Automation of Landmark Identification Using the You Only Look Once Machine Learning Algorithm

**FIGURE 2:**
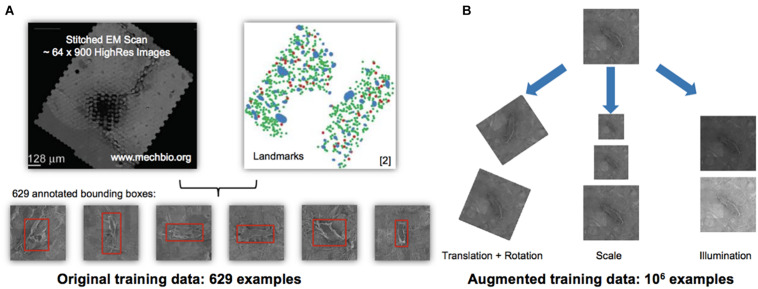
Automated detection algorithm to classify osteocytes using manual methods and the You Only Look Once algorithm (YOLO; Redmon et al., 2015). **(A,B)** Training and testing data for the machine learning algorithm using the 1st generation mSEM map. The manually acquired training data set, comprising 629 examples of osteocytes (**A**—stitched electron microscopy scan on left and manually located and “pinned,” using Google Maps API, osteocytes, where green indicates viable and red marks pyknotic), was scaled up to an augmented training set of 10^6^ examples **(B)** using digital permutations of translation and rotation, scale and illumination. From the augmented dataset, 75% of the data was used for training and 25% of the data was used for testing of the YOLO algorithm, all using the data from the 1st generation map. The algorithm was then run independently on the 2nd (Figure 3) and 3rd generation maps. Note: the red bounding boxes **(A)** indicate detected cells prior to classification, i.e., not indicative of cells’ health status.

Increasing dataset sizes necessitated development of objective, automated methods for identification and quantification of cells, an ideal application for machine learning approaches. To this end, we implemented the so-called “You Only Look Once” (YOLO) machine learning algorithm (Redmon et al., 2015), using the previously marked datasets as training data (Figures 2A,B). Seventy five percent of the augmented dataset was used to train the model and 25% of the data were set aside to test the model. The You Only Look Once machine learning algorithm was applied to detect cells on all datasets (Figure 2C). The image processing was simple and straightforward. The YOLO detection system resizes the input image to 448 × 448, runs a single convolutional network on the image, and thresholds the resulting detections by the model’s confidence.

**FIGURE 3:**
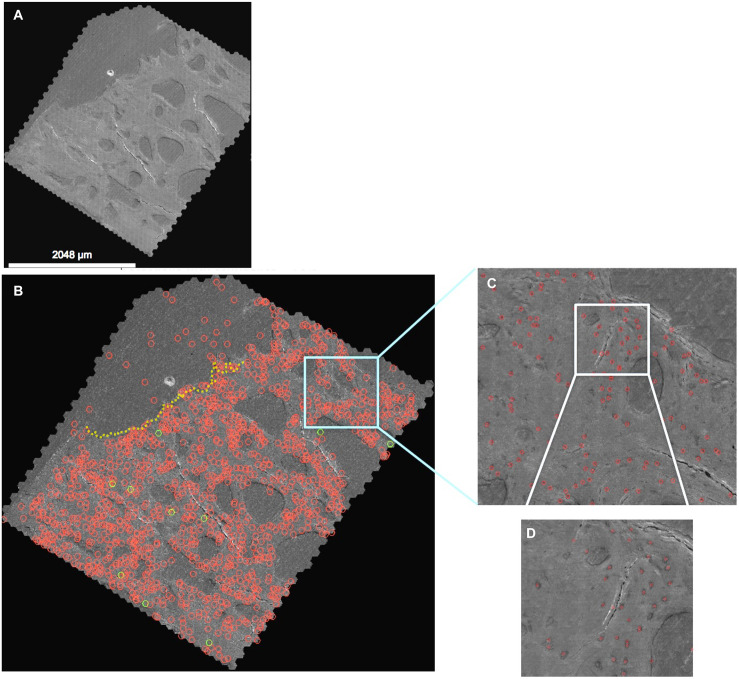
Automated detection algorithm applied to the 2nd generation map **(A)**, where detections with greater than 70% confidence (quantitative prediction of match to “ground truth” defined by training data) are depicted. **(B)** The entire map, depicting higher resolution details at increasing levels of zoom **(C,D)** in the Google Maps API. Note: the red circles indicate detected cells which are changed to green when the classifier (three or more processes) is met. Red circles above the dotted yellow line delineating the edge of the tissue surface (**B**, upper corner) are cells, vessels and artifacts outside of the femoral head and neck tissue. The map depicted here can be navigated and explored like Google Maps at http://www.mechbio.org/sites/mechbio/files/maps7/index.html to access the map, type in user: mechbio, password: #google-maps.

In summary, we acquired three datasets rendering osteocyte networks in tissues of the femoral head. The different datasets include data from different samples imaged using new generations of mSEM and associated increasing computational capacity. The first data set included 5.69 mm^2^ tissue and over 50,000 images (0.08 terabyte), with manual identification of 629 osteocytes taking several weeks’ time. To enable automated, rapid detection of osteocytes and in consideration of the increasing size and complexity of the datasets enabled through advances in the mSEM instrument and parallel computational advances (second and third generations) over the past decade (from an advanced prototype to a commercial system), we applied a machine learning algorithm to detect osteocytes based on the You Only Look Once (YOLO) convolutional neural network described originally by Redmon et al. (2015). The YOLO algorithm is faster and more efficient than typical classifier-based algorithms. YOLO “looks at an image once” (thus the acronym) to predict the presence of objects and their locations. The image is divided into an S × S grid in which bounding boxes and confidence scores for object detection within bounding boxes are calculated. The confidence scores give a quantitative probability of how similar the predicted box detecting an object is with the training data (ground truth). The higher the confidence score, the more accurate the prediction (Redmon et al., 2015; Shivaprasad, 2019).

We applied the machine learning approach to second and third generation maps, with the third data set comprising 1,810 mm^2^ tissue and over 7,000,000 images (10.98 TB), identifying a total of 206,180 osteocytes in 100 h on a graphics processing unit (GPU, GeForce GTX 1080 graphics card enabled) compared to the manual pinning method of our previously published work (Knothe Tate et al., 2016c) that identified 629 osteocytes manually over several weeks. The algorithm performance currently exceeds 92% accuracy for osteocyte detection and classification, based on the accuracy of detecting the 629 original cells using the trained procedure.

Osteocyte coordinates can be extracted from the YOLO classified image set, enabling high throughput analyses of massive datasets, which in the future could include other cellular inhabitants of tissues including blood cells, immune cells, chondrocytes, etc. While the method shows great promise for automated detection of cells, the greatest limitation of the method is the definition of appropriate and unbiased classifiers. The definition of osteocytes as pyknotic and viable based on the number of cell processes was shown to be flawed in a parallel study testing the assumption using biochemical based viability measures (Anastopolous and Knothe Tate, 2021). Not only did the method not account for empty osteocyte lacunae that appeared as “ghost osteocytes” (resin filled empty lacunae) but also osteocyte process number has not been tied inextricably to cell viability. Multimodal imaging methods and assays using iodine to stain nuclear material demonstrate that better descriptors of cell health are needed (Anastopolous and Knothe Tate, 2021).

With these limitations in mind, the technological approach provides novel opportunities for a new field of cellular epidemiology, where emergent changes in cell health may in the future be used to predict disease outbreaks and prevent disease transmission, much like they are used at the length scale of human inhabitants of geographically defined environments (Knothe Tate et al., 2016c; Dong et al., 2019). The described workflow and data analytics pipeline enables acquisition, preparation, and imaging of tissue and organ samples, as well as post-imaging rendering of and analysis of cellular networks from different tissues across length scales, of nano- to meso-length scales.

### Implications for Understanding Neuromechanics and the Control of Physical Behavior and Neuromuscular Training

In addition to its obvious application for development of next generation materials, devices and diagnostics, this disruptive biosystems engineering platform provides a novel tool for elucidating the relationship between neural and musculoskeletal connectomics, movement, navigation and memory (Epstein et al., 2017; Knothe Tate, 2017). The loss in connectivity observed in the dendritic osteocyte network of aging and diseased bone is similar to that of the brain cells in aging individuals and patients with early onset dementia (Huang et al., 2015; Knothe Tate and Fath, 2016; Knothe Tate et al., 2016c; Pereira et al., 2016; Rossini et al., 2020). The possibility that movement and geographical maps are encoded not only in the brain but also in the musculoskeletal tissues is tantalizing. The technological platform here provides a means by which networks within tissues and organs of different systems within individual organisms can be studied, from cellular to whole being contexts. This is expected to lead to discovery of novel mechanisms underpinning motor neural circuitry and biomechanical action. Just as dogs and other mammals train their neural networks in their sleep, running and jumping across virtual dream terrains, perhaps the physical experience of life itself is encoded in our cells, the structural proteins our cells manufacture via gene expression and secrete to form our tissues, as well as the adaptation of our tissues throughout life (Knothe Tate, 2020).

## Materials and Methods

### Sample Preparation

Tissues were fixed in a combination of 4% formaldehyde and 2.5% paraformaldehyde in 0.2 M cacodylate buffer. Tissues were then embedded in poly(methyl methacrylate) under vacuum (Ngo et al., 2019). Following this, the sample was precision CNC-milled and etched using 0.02 M hydrochloric acid and 10% sodium hypochlorite to remove organic and inorganic top layer, in order to reveal cellular material (Reilly et al., 2001; Knapp et al., 2002). The sample was then carbon coated and placed under a vacuum, preparing for mSEM imaging. Imaging was performed on the 61-beam Zeiss MultiSEM 505-prototype at 12 nm pixel size.

### Identification of Relevant Landmarks, Creation of Training Datasets

Manual marking of landmarks was described in a previous paper (Knothe Tate et al., 2016c) and the thereby identified 629 osteocytes were used as the basis for a training and testing data set (Figure 2).

### Machine Learning Approach Using the You Only Look Once (YOLO) Algorithm

The “You Only Look Once” (YOLO) neural network automated object detection algorithm (Redmon et al., 2015; *described further for the layperson in* Shivaprasad, 2019) was applied to facilitate rapid throughput diagnostic assessment of imaging datasets while mitigating the effects of observer bias. An image is first divided into a grid, where each grid cell predicts bounding boxes for objects. Then probability-based confidence scores are calculated for the bounding boxes; the confidence score compares how close the predicted bounding box and object therein matches the known objects and bounding boxes defined by the training data set, or “ground truth.” To prevent multiple detections of the same object, the bounding box with highest confidence greater than 0.5 (which would be 50% chance of matching the training data point) is selected from overlapping bounding boxes; referred to as non-max suppression, this process results in highest confidence for model predictions per object detected and maximizes accuracy of the model.

**FIGURE 4:**
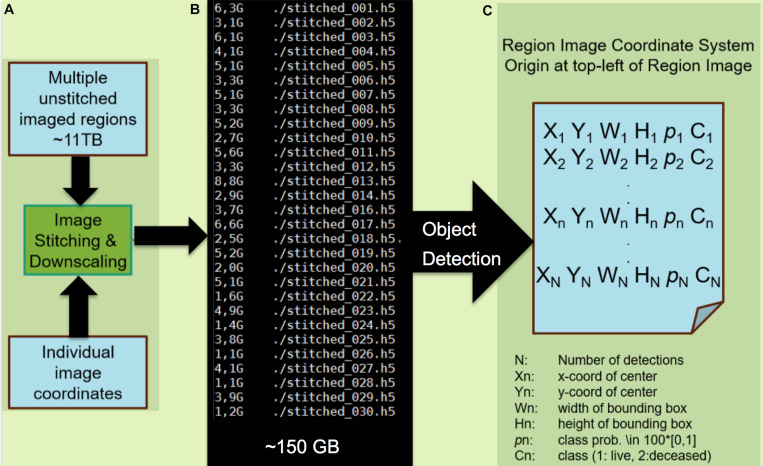
Processing of images and application of the machine learning classification algorithm. **(A)** Module A preprocesses the mSEM output by stitching individual images into region-wide panoramas by the virtue of recorded image coordinates. In the interest of computational efficiency, the resulting image is down-scaled such that individual cells occupy *circa* 200 × 200 pixels. Here, the 11TB mSEM output from the 3rd generation map is stitched into 30 image regions amounting to 150 GB of data after downscaling. **(B)** Module B applies the pretrained object detector. **(C)** The output is a file listing that includes the location of each detected cell as a bounding box (X,Y,W,H), class (viable/pyknotic, annotated as “deceased”) and associated confidence *p*. Each of N detections results in a set of five predictions for each corresponding bounding box, including the x and y coordinates of the center of the bounding box (X_*n*_,Y_*n*_), width and height of the bounding box (W_*n*_,H_*n*_), and the confidence of the detection (*p*_*n*_ where a confidence or probability of 0 means no object was detected and 1.0 means a perfect match with “ground truth”). The object is then further classified as live or deceased (C_*n*_). The object detector is pretrained using 600 living and 50 pyknotic examples (Knothe Tate et al., 2016b) and took 12 h to train on a single graphics processing unit (GTX1080). The testing phase on the 150 GB dataset lasted 100 h. Note: modules A and B can be combined into a single module.

Initially, YOLO was trained for automated osteocyte detection, using 629 annotated cells, which were further augmented to 10^6^ examples through variation by rotation, scale and contrast (Figure 2). Unseen images were then processed with YOLO and automatically detected objects were identified and marked by bounding boxes. The success of the YOLO algorithm has been proven for detecting osteocytes in the 2nd generation map within 100 h of testing. A straight-forward approach to improve the detector performance includes collection of more than 1,000 false- and missed-detections (Figure 4) to obtain a more representative training dataset.

## Data Availability Statement

The original contributions presented in the study are included in the article/supplementary material, further inquiries can be directed to the corresponding author/s.

## Ethics Statement

The studies involving human participants were reviewed and approved by the Cleveland Clinic Institutional Review Board.

## Author Contributions

MK and DZ conceived the work. The specimens were collected and prepared for imaging by MK with assistance from the Cleveland Clinic and Zeiss teams. Image acquisition was carried out by the Zeiss Team, led by DZ and MK in the Demonstration Labs at Carl Zeiss Microscopy GmbH in Oberkochen. The machine learning algorithm was developed and tested by AS and CW. The manuscript was written by MK and was revised and approved of by all coauthors. All authors contributed to the article and approved the submitted version.

## Conflict of Interest

Zeiss provided in kind support for this project, which is of a fundamental and translational nature. AS and CW were employed by Zeiss AG. DZ was employed by Carl Zeiss MultiSEM GmbH. The remaining author declares that the research was conducted in the absence of any commercial or financial relationships that could be construed as a potential conflict of interest.
